# Variable Responses to CFTR Correctors *in vitro*: Estimating the Design Effect in Precision Medicine

**DOI:** 10.3389/fphar.2018.01490

**Published:** 2018-12-19

**Authors:** Elizabeth Matthes, Julie Goepp, Carolina Martini, Jiajie Shan, Jie Liao, David Y. Thomas, John W. Hanrahan

**Affiliations:** ^1^Department of Physiology, McGill University, Montréal, QC, Canada; ^2^Cystic Fibrosis Translational Research Centre, McGill University, Montréal, QC, Canada; ^3^Department of Biochemistry, McGill University, Montréal, QC, Canada; ^4^Research Institute of the McGill University Health Centre, McGill University, Montréal, QC, Canada

**Keywords:** precision medicine, cystic fibrosis, correctors, lumacaftor, Orkambi, group sampling, design effect, power calculations

## Abstract

Interest in precision medicine has grown in recent years due to the variable clinical benefit provided by some medications, their cost, and by new opportunities to tailor therapies to individual patients. In cystic fibrosis it may soon be possible to test several corrector drugs that improve the folding and functional expression of mutant cystic fibrosis transmembrane conductance regulator (CFTR) prospectively using cells from a patient to find the one that is best for that individual. Patient-to-patient variation in cell culture responses to correctors and the reproducibility of those responses has not been studied quantitatively. We measured the functional correction provided by lumacaftor (VX-809) using bronchial epithelial cells from 20 patients homozygous for the F508del-CFTR mutation. Significant differences were observed between individuals, supporting the utility of prospective testing. However, when correction of F508del-CFTR was measured repeatedly using cell aliquots from the same individuals, a design effect was observed that would impact statistical tests of significance. The results suggest that the sample size obtained from power calculations should be increased to compensate for group sampling when CFTR corrector drugs are compared *in vitro* for precision medicine.

## Introduction

Cystic fibrosis (CF) is a relatively common orphan disease caused by loss-of-function mutations in the gene encoding CFTR (cystic fibrosis transmembrane conductance regulator), a tightly regulated anion channel (Riordan, 2008). CFTR mediates secretion across many epithelia in the body and is required for efficient mucociliary clearance of inhaled pathogens from the lungs (Ratjen et al., 2015). CF modulators such as lumacaftor (VX-809) that partially correct the misfolding and/or potentiate the activity of mutant CFTR channels are available and more are in the pipeline, however, they are expensive and their clinical benefit varies between individuals (Boyle et al., 2014; Hanrahan et al., 2017). With precision medicine one could potentially test multiple drugs on cells from a patient to identify the one that is most efficacious for that individual.

Although CF seems ideally suited for applying the precision medicine approach (Amaral, 2015; Martiniano et al., 2016; Burgener and Moss, 2018; Cholon and Gentzsch, 2018), some practical issues remain to be addressed. Foremost among these is whether differences in functional correction measured using primary cultured cells from different patients are statistically significant. This is obviously essential if prospective testing *in vitro* is to be useful for making prescribing decisions. The variable clinical benefit provided by Orkambi^®^ is well known (Boyle et al., 2014), however, it remains unclear if similar variability exists at the level of epithelial cells and persists in cell culture. If cells from different patients vary in their response to drugs and the responses correlate with clinical benefit, a practical question arises as to how many assays would be needed to conclude that one corrector is more effective than another for that individual. The causes of variability are not yet understood therefore it is not possible to develop a statistical model capable of predicting responses to a drug. Nevertheless, methods for collecting and analyzing data are needed if efficacy in cell-based assays is to be useful for precision medicine.

Assays of CFTR function that utilize different cell types have been developed and could potentially be used to test the drug responsiveness of individual patients. Rescue of the mutant CFTR can be assayed directly by measuring Cl^−^ transport across tissue samples or primary cell cultures using electrophysiology (Van Goor et al., 2011; Brewington et al., 2018). CFTR function can also be assayed indirectly by measuring net fluid transport across intestinal organoids prepared from rectal biopsies (Dekkers et al., 2013, 2016) or airway epithelial spheroids prepared from cells that are obtained by brushing or curettage of the nasal or bronchial mucosa (Guimbellot et al., 2017; McCarthy et al., 2018). Induced pluripotent stem cells (iPSCs) may be useful if they can be made to differentiate fully and recapitulate variable drug responses between patients having the same CFTR genotype, which may depend on both genetic and epigenetic factors. Correlations have recently been reported when assaying CFTR function in the nasal epithelium *in vivo* and in cultured cells from patients with different CFTR mutations (Pranke et al., 2017). Similar responses have also been observed in nasal and bronchial epithelial cultures (Pranke et al., 2017; Brewington et al., 2018). Importantly, for seven patients homozygous for F508del-CFTR there was a correlation between mean functional rescue in nasal cell cultures and the clinical response to Orkambi measured as % FEV1 (% predicted forced expiratory volume in 1 s) (Pranke et al., 2017).

In this paper we start by quantifying variability in functional correction amongst patients having the same genotype (F508del-CFTR/F508del-CFTR) using well differentiated bronchial epithelial cell cultures. Then we explore the reproducibility of correction by repeatedly testing samples from large pools of cells from two individuals, analogous to sampling the airway *in vivo.* We observe a design effect caused by group sampling that needs to be considered when testing the statistical significance of differences in correction.

## Materials and Methods

### Cells

**FIGURE 1:**
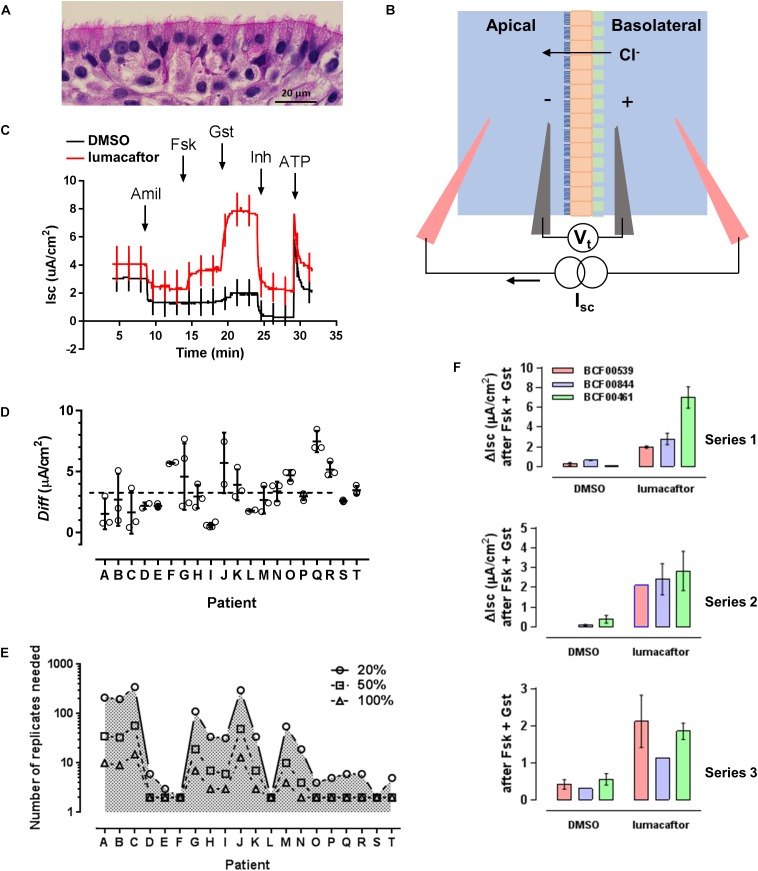
An assay for testing F508del-CFTR corrector drugs. **(A)** Well differentiated primary human bronchial epithelial (HBE) cells cultured at the air-liquid interface. **(B)** Electrophysiological measurement of CFTR function as short-circuit current (I_sc_), the current needed to clamp the transepithelial voltage (V_t_) at 0 mV. Epithelial cells (orange) cultured on a porous support (green) are mounted in modified Ussing chambers. A basolateral-to-apical Cl^−^ gradient is imposed to generate a secretory flux through rescued mutant CFTR channels. **(C)** Representative recordings of cells pretreated for 24 h with DMSO (vehicle) or lumacaftor (corrector), then exposed sequentially to Na^+^ channel blocker amiloride (10 μM Amil, apical), forskolin (10 μM Fsk, bilateral, activator), genistein (50 μM Gst, apical, potentiator), CFTR_inh_-172 (10 μM Inh, apical, CFTR inhibitor), and ATP (10 μM, apical, purinergic agonist to stimulate Ca^2+^-activated Cl^−^ channels as a positive control for viability). Current deflections show responses to brief voltage steps to +/−1 mV to monitor transepithelial resistance. **(D)** Response of cells from 20 patients to lumacaftor shown as the difference (*Diff*) in ΔI_sc_ stimulated by forskolin + genistein when cells were pretreated with lumacaftor or DMSO. (means +/− s.d., *n* = 3 for each condition). **(E)** Predicted number of replicates needed to detect a 20, 50, and 100% change in correction, calculated for each patient. **(F)** Three series of assays performed independently on the same three patients under identical conditions. In each series, cells from the same patients were exposed in triplicate to vehicle (DMSO) or corrector (lumacaftor).

**FIGURE 2:**
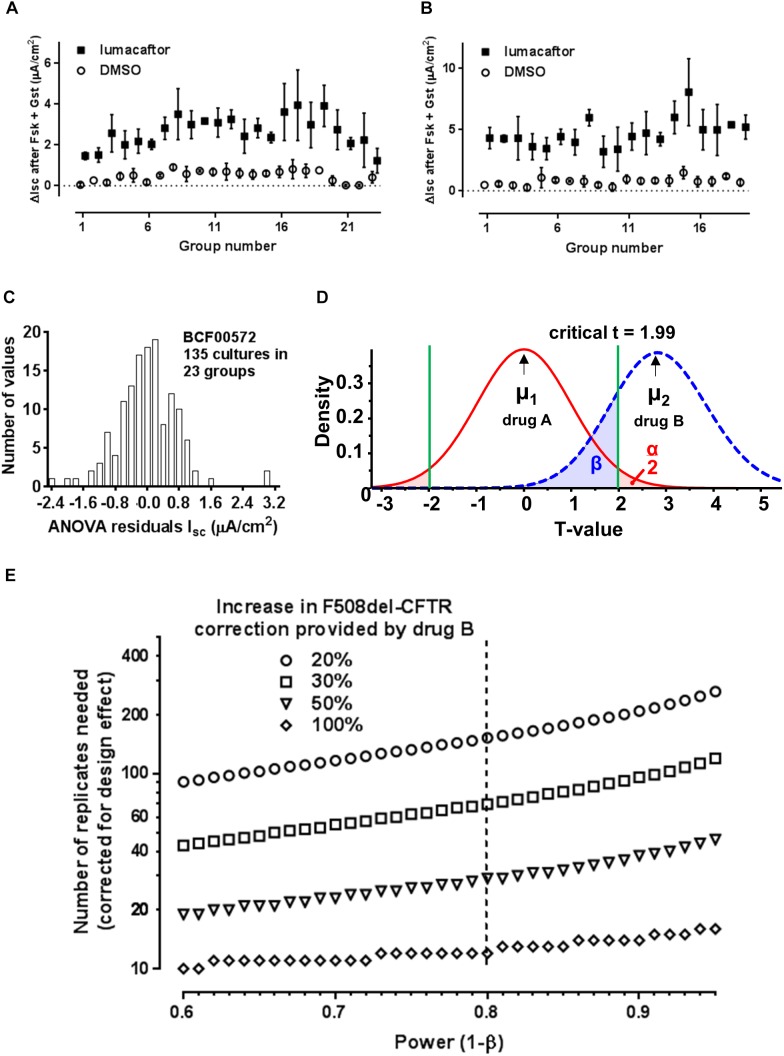
Impact of group sampling when comparing two correctors. **(A)** Simulation of group sampling by measuring F508del-CFTR functional correction in aliquots of the same cell population (patient BCF00572). Response to forskolin + genistein after treatment with vehicle (open circles) or corrector (closed squares) in 23 groups of 3–9 cultures (technical replicates). **(B)** Same as panel **A** but with cells from patient BCF00710. **(C)** Distribution of the residuals from one-way random effects ANOVA of lumacaftor treated cell cultures from patient BCF00572. **(D)** Predicted *t*-distributions for two correctors, where hypothetical drug B gives a mean F508del-CFTR correction μ_2_ that is 20% higher than corrector A (μ_1_). The calculations assume α = 0.05, β = 0.8, mean response to forskolin + genistein after pretreatment with corrector A = 3.3 μA/cm^2^, and s.d. = 1.0 μA/cm^2^ for both drugs. **(E)** Number of replicates needed to detect a significant change in correction by drug B compared to drug A, assuming there is a real improvement of 20, 30, 50, or 100%, after compensation for the design effect due to group sampling. The number of replicates is shown for power 0.60–0.95 (i.e., probability of a false negative of 0.4–0.05).

Primary human bronchial epithelial (HBE) cells were obtained from the Primary Airway Cell Biobank at the McGill CF Translational Research centre (CFTRc) and cultured at the air liquid interface as described previously (Fulcher et al., 2005; Robert et al., 2007). Briefly, lung tissue from patients undergoing lung transplantation was obtained from the Biobank of respiratory tissues at the Centre Hospitalier de l’Université de Montréal and Institut de recherche cliniques de Montréal. Informed, written consent was obtained and all procedures were approved by the Institutional Review Board of McGill University (#A08-M70-14B) and followed Canadian Institutes of Health Research guidelines. Cells were isolated from bronchial tissue by enzyme digestion and cultured in bronchial epithelial growth medium (BEGM) on type I collagen-coated plastic flasks (Vitrogen 100, PureCol; Advanced BioMatrix), then trypsinized, counted, and used immediately for experiments (Figures 1D,F) or cryopreserved in aliquots of 2 million cells and used within 18 months (Figures 1F and 2A,B). Bronchial epithelial cell growth medium (BEGM) was used during cell isolation and initial culture of the cells (i.e., passage 0, P0). BEGM consists of Laboratory of Human Carcinogenesis (LHC) Basal Medium (Invitrogen) and ∼20 supplements including bovine serum albumin (BSA) but not serum [see (Fulcher et al., 2005) for details]. Once cells had been thawed and seeded on porous supports they were cultured using air-liquid interface (ALI) medium to induce differentiation. The ALI medium was a 50:50 mixture of LHC Basal Medium and Dulbecco’s modification of Eagle medium (D-MEM) with less added human epidermal growth factor (hEGF) but other supplements including BSA at similar levels to BEGM. Drug treatments were performed for 24 h in ALI medium without BSA or antibiotics.

Freshly isolated or thawed cells were seeded on collagen coated 6.5 mm Costar^®^ 0.4 μm pore, polyester membrane inserts (Corning) and grown under submerged conditions for 4 days. The apical medium was then removed and cells were allowed to differentiate at the ALI for 4 weeks before use in correction assays. Isolation and growth media were supplemented with antibiotics that were selected based on recent patient microbiology reports. Only penicillin and streptomycin were added to ALI cultures. Cells were used at first passage (P1), and comparisons between patients used recently isolated cells that had not been frozen. Repeated sampling of two patients was performed using cells from large isolates that had been cryopreserved in aliquots of 2 million cells (Fulcher et al., 2005; Robert et al., 2010). Mature cultures were pseudostratified and appeared highly differentiated with ciliated, goblet and basal cells (Figure 1A).

### Corrector Treatment and CFTR Functional Assays

When comparing different patients, three well differentiated monolayers were pretreated for 24 h at 37°C with vehicle (0.1% DMSO dimethyl sulfoxide) and three were pretreated with lumacaftor (1 μM; Selleck Chemicals, Houston, TX, United States) and the same final concentration of DMSO. The six monolayers were then mounted in modified Ussing chambers and short-circuit current (I_sc_ in μA cm^−2^) was measured to assay CFTR function in all six cultures simultaneously (Figure 1B). Transepithelial voltage was clamped at 0 mV except for 2 s bipolar pulses to ±1 mV at 100 s intervals to monitor transepithelial resistance. A basolateral-to-apical NaCl chloride gradient was imposed to increase the signal-to-noise of the current response; apical (in mM): 1.2 NaCl, 115 Na-gluconate, 25 NaHCO_3_, 1.2 MgCl_2_, 4 CaCl_2_, 2.4 KH_2_PO_4_, 1.24 K_2_HPO_4_, 10 Glucose; basolateral (in mM): 115 NaCl, 25 NaHCO_3_, 1.2 MgCl, 1.2 CaCl_2_, 2.4 KH_2_PO_4_, 1.24 K_2_HPO_4_, 10 Glucose. After the short-circuit current had stabilized (typically 2–3 min), Fsk (10 μM) was added to both sides to raise intracellular cAMP. This was followed by sequential additions of the potentiator genistein (Gst, 50 μM), the CFTR inhibitor CFTR_inh_-172 (10 μM) and the purinergic agonist ATP (100 μM) to the apical side to stimulate Ca^2+^-activated Cl^−^ channels as a positive control and to confirm cell viability (Matthes et al., 2016). Potentiation by 50 μM genistein was similar to that produced by 0.1 μM ivorcaftor (VX-770 or Kalydeco^®^) and was used because it was more easily washed from the chambers. The stimulation of I_sc_ (taken as the increase from the steady-state baseline level before adding forskolin to the maximum current after genistein addition; ΔI_sc_) was used to measure of F508del-CFTR functional expression (Figure 1C). Assays using CF mouse intestine were performed as described previously (Robert et al., 2010). Rotterdam delF508/delF508-CFTR mice (*Cftr*^*tm*1*Eur*^), FVB inbred, 14–17 weeks old, 24–30 g) were used (van Doorninck et al., 1995; Scholte et al., 2004). All procedures followed Canadian Institutes of Health Research guidelines and were approved by the faculty Animal Care Committee at McGill University (#2012-7119). The ileum was stripped of muscle and mounted in mini-Ussing chambers (Physiological Instruments, San Diego, CA, United States). Tissues were bathed with William’s E-Glutamax (x1; Gibco) supplemented with insulin (10 μg/ml) and dexamethasone (20 μg/ml). Short-circuit current and resistance were measured before and after sequential additions of forskolin (10 μM) and genistein (50 μM) to the apical side. After steady-state stimulation these were washed from the chambers and lumacaftor or vehicle (0.1% DMSO) was added for 4 h. Forskolin and genistein were then re-assayed and the difference between the I_sc_ responses before and after exposure to lumacaftor or vehicle (which served as a time control) were used as a measure of correction. Reagents were from Sigma unless otherwise indicated.

#### Statistics

Results are expressed as the mean ± S.E.M. of *n* observations. F508del-CFTR correction was displayed as *Diff* (the difference between maximum *I_sc_* response to forskolin + genistein when cells were pretreated with lumacaftor vs. DMSO

(1) $$\mathrm{Diff}=\Delta {\mathrm{Isc}}_{\mathrm{treated}}-\bar{\Delta I}{\mathrm{sc}}_{\mathrm{ctrl}}$$

Although the same number of cultures was exposed to drug and to vehicle, the measurements were unpaired and all three DMSO controls were equally applicable to each drug-treated culture. *Diff* was calculated and plotted for clarity to show correction but was not used in statistical tests, which were based on the raw data collected under each condition.

The impact of group sampling was determined as the intraclass correlation coefficient (ICC, also called intracluster correlation coefficient or “rho” ρ) the ratio of the variance between groups to the sum of variances between and within groups (Donner and Klar, 2000; Killip et al., 2004). ρ can range from 0 when there is no effect of group sampling to 1 when the replicates within groups are perfectly consistent and variation is entirely between groups. The results of ANOVA were then used to determine ρ(Δ*Isc _treated_*) as:

(2) $$\rho =\frac{{\mathrm{MS}}_{B}-{\mathrm{MS}}_{W}}{{\mathrm{MS}}_{B}+(n_{o}-1){\mathrm{MS}}_{W}}$$

where *MS_B_* and *MS_W_* are the mean squares between and within groups, respectively, and η_o_ is the average number of replicates per group calculated as:

(3) $$\frac{\left(\mathrm{total}\;\#\;\mathrm{of replicates}-\mathrm{sum of the squared}\;\#\;\mathrm{of replicates in each group}/\mathrm{total}\;\#\;\mathrm{of replicates}\right)}{\mathrm{total}\;\#\;\mathrm{of groups}-1}$$

Residuals for 22 of the 23 group samples passed the Kolmogorov-Smirnov normality test with α = 0.05 using GraphPad Prism v. 6.04 (GraphPad Software, La Jolla, CA, United States) ^1^. When the more stringent D’Agostino-Pearson K^2^ omnibus test (D’agostino et al., 1990) was applied to groups having *n* ≥ 9 (the minimum needed) they passed the normality test. When residuals for samples from all groups were pooled there was deviation from normality due to positive kurtosis, however, the distribution was symmetric and well fitted by a Gaussian curve (*r*^2^ = 0.9944) after excluding two bins as outliers (Figure 2C). The robustness of ANOVA when there are moderate departures from normality (Motulsky, 2014) suggests the ANOVA results are valid. The standard deviation of the I_sc_ response to drug _(Δ*Isc _treated_*)_ was used in the program G^∗^Power v3.9.1.2 when estimating the number of replicates needed to determine if lumacaftor causes significant correction for each of the 20 patients and for different effect sizes (Faul et al., 2007).

## Results

Cystic fibrosis bronchial epithelial cells from all 20 patients were more strongly stimulated by forskolin + genistein after 24 h pretreatment lumacaftor (Figure 1D). The average stimulation after treatment with lumacaftor was 11.4% that measured in non-CF cells expressing wild-type CFTR (pooled data from 11 non-CF donors). This is similar to a previous report in which cells from seven patients that had been cultured in medium supplemented with Ultroser G and assayed acutely using a different potentiator (Van Goor et al., 2011).

The I_sc_ responses were ∼15-fold more variable after drug pretreatment than after pretreatment with vehicle. The variation was quantified using a mixed, two-way analysis of variance (ANOVA) of all data in both groups (i.e., baseline I_sc_ and maximum I_sc_ after stimulation by forskolin + genistein). Most (51.0%) of the variability was associated with the effect of drug pretreatment while 19.3% could be attributed to patients and 11.9% was explained by interaction between these factors; i.e., patient-to-patient differences in the response to lumacaftor (parameter of interest for precision medicine). Despite the small sample size, the null hypothesis that cells from different patients respond similarly to lumacaftor could be rejected with *P* = 0.0001. This is consistent with a previous study using a linear random effects model in which most variability in the response to lumacaftor was due to inter-patient rather than intra-patient variability (Pranke et al., 2017). Our power calculations to estimate the number of replicates needed for lumacaftor responses to reach statistical significance for a given effect size (i.e., increase of 20, 50, or 100%) yielded variable results when based on triplicate assays (Figure 1E) suggesting a larger sample size is needed to reliably estimate the standard deviation. Nevertheless, the results provide support for precision medicine in CF in that they show significant variability in drug responses between airway epithelial cells cultured from different individuals.

To assess the reproducibility of F508del-CFTR correction we compared the stimulation by forskolin + genistein on 3 successive weeks using different ALI cultures. The cells were prepared from different patients, cryopreserved and then thawed at 1 week intervals and cultured under identical conditions for 1 month for assays. Cells from one patient responded similarly to forskolin + genistein after pretreatment with lumacaftor in all three sets of experiments. However, ΔI_sc_ was more variable when cells from the other two patients (Figure 1F).

To explore this variability further we simulated *in vivo* sampling by dividing a pool of 170 million HBE cells from one patient into aliquots of 2 million. Each aliquot was considered a group sample from the airway surface epithelium to mimic repeated collection of small samples *in vivo* by bronchial brushing, but under well controlled conditions. Multiple ALI cultures were prepared using 23 randomly chosen aliquots (2–18 cultures per aliquot) and the ΔI_sc_ response to forskolin + genistein was measured using equal numbers of vehicle- and lumacaftor-treated replicates from each aliquot. Cells that had been treated with lumacaftor were always more responsive to forskolin + genistein than DMSO controls as expected (Figure 2A). One-way random effects ANOVA of ΔI_sc_ revealed significant variation between the groups (*P* < 0.0001), despite coming from the same original pool of cells.

Since prospective testing for the response to a CF drug will likely involve analyzing small cell samples *in vitro*, we examined the statistical consequences of such group sampling on correction. We calculated the intraclass correlation coefficient (ICC, also called ρ rho), which is the ratio of the variance between groups to the sum of the variances between and within groups. This was done when correction by lumacaftor was assayed repeatedly using 135 cultures from one patient (BCF00572). On average, each group consisted of η_o_ = 5.86 replicates and had ρ = 0.377, yielding a design effect [1 + ρ(η_o_−1)] = 2.83. ICC was somewhat higher for vehicle-treated cells (ρ = 0.4466), in agreement with the lower variability of DMSO controls in Figure 2A.

To determine if the design effect is observed generally when assaying F508del correction we analyzed cells from a second patient (BCF00710). We selected 19 aliquots of cells (2 million cells each) at random from a pool of 280 million cells, cultured them at the ALI for 1 month, and assayed their stimulation by forskolin + genistein with and without lumacaftor pretreatment as before (Figure 2B). In this experiment there were η_o_ = 4.34 replicates per lumacaftor treatment group and equation (2) yielded ρ = 0.2816 and a design effect of 1.94. Plotting the residuals from a one-way random effects ANOVA of all data from lumacaftor-treated cultures from patient BCF00572 gave a bell-shaped frequency distribution that showed positive kurtosis but only moderate deviation from normality, supporting the validity of ANOVA (Figure 2C). In summary, the results from both patients indicate that variability of drug responses measured using group samples underestimates the true variation in the original population. Correcting for the design effect is necessary when comparing drug responses *in vitro* for precision medicine; two- to three-fold more replicates than estimated by regular power calculations are needed to draw conclusions based on two-tailed *t*-tests.

With this in mind we asked “How many replicates are needed to conclude that corrector B is more effective than corrector A using cells from one individual that have been collected and cultured *in vitro*?” To answer this we assumed a typical stimulation by forskolin + genistein after pretreatment with corrector A (lumacaftor; ΔI_sc_ = 3.3 μA/cm^2^, s.d. = 0.962 μA/cm^2^; the mean from 20 patients studied under our conditions). This response was compared to that generated by hypothetical “corrector B,” which was assumed to have the same variance as lumacaftor. We set α = 0.05 (probability of Type I error or false positive when testing the null hypothesis *H*_o_ that correctors A and B have the same efficacy) and β = 0.2 (probability of a Type II error or false negative, corresponding to a statistical power of 1-β = 0.8; see Figure 2D). The number of replicates needed was then calculated as a function of power when corrector B would provide a real improvement in F508del-CFTR correction that is 20, 30, 50, or 100% higher than corrector A. Replicates were assumed to be independent samples, then the predicted number of replicates was then multiplied by 2.83 to correct for the effect of group sampling (Figure 2E).

The results indicate that if hypothetical corrector B has twice the efficacy of corrector A (i.e., increases F508del-CFTR rescue by 100%), 12 replicates from a group sample would be sufficient to conclude there is a significant difference between correctors. However, more replicates are needed if the real change in efficacy is less dramatic, e.g., 153 when corrector B increases F508del-CFTR function by only 20%.

## Discussion

The present results demonstrate statistically significant variation in responses to lumacaftor when assayed using cultured cells from different patients. Variable corrector responses have been observed previously (Van Goor et al., 2011; Eckford et al., 2014) and were analyzed for homozygous F508del CFTR patients in one study (Pranke et al., 2017), however, the implications for comparing efficacies of different drugs have not been explored. We also found considerable variability between cell samples from the same patient and explored its consequences when distinguishing between correctors. Prospective drug testing *in vitro* will likely begin with the collection of a small sample of epithelial cells from the patient. We used bronchial epithelial cells, however, similar results would be expected when small numbers of epithelial cells are harvested from the nasal or bronchial mucosa or by rectal biopsy. Since group samples have reduced standard deviation compared to the original epithelial cell population, they are expected to cause underestimation of the sample size needed for statistical testing, although this estimate can be corrected by determining the design effect.

Air-liquid interface cultures are considered to be the “gold standard” for testing CFTR modulators and have been accepted by the FDA when evaluating new drug applications (Durmowicz et al., 2018). Using this model to assay samples from a single large population of airway cells we estimated the design effect to range between 2 and 3. The sources of variability between group samples and replicates within groups are unknown and may reflect heterogeneity in the cell isolate and/or subtle differences during differentiation in prolonged culture. Cells were handled identically according to detailed standard operating procedures, nevertheless we cannot exclude slight variations in cell viability after thawing that could affect seeding density, or volume when the cells were fed with fresh medium. Cells were treated with corrector for 24 h in our study, however, lumacaftor is hydrophobic and longer exposures (48–72 h) are expected to increase its uptake and might reduce variability. The efficacy of Orkambi appears to be limited mainly by the modest efficacy of lumacaftor, therefore we have focussed on it in this study and pretreated cells only with lumacaftor. Simultaneous exposure to another drug such as ivacaftor could increase variability since variances add, although it will be important to test prolonged exposure to ivacaftor at clinically-relevant, low nanomolar free concentrations to avoid adverse effects on CFTR functional rescue by lumacaftor (Matthes et al., 2016). When multiple corrector drugs become available for CF they may be compared *in vitro* for precision medicine on the assumption that more functional expression *in vitro* will correlate with better symptoms *in vivo*. Such a correlation is observed between mean responses of multiple homozygous F508del-CFTR patients to Orkambi *in vivo* and nasal epithelial cultures from the same patients treated with ivorcaftor + lumacaftor (Pranke et al., 2017). Those results are also consistent with the ability of ivorcaftor alone to increase Cl^−^ conductance in cells expressing G551D-CFTR but not F508del-CFTR (Van Goor et al., 2009) and to improve lung function in patients with G551D (Accurso et al., 2010) but not in F508del homozygotes (Flume et al., 2012). Why correction varies between patients with the same CFTR genotype remains uncertain. Variation in genetic background (i.e., genes other than CFTR) may contribute since genome wide association studies have identified several genes that can modify CF disease severity (Cutting et al., 1990; Wright et al., 2011; Sun et al., 2012; Blackman et al., 2013; Corvol et al., 2015). Epigenetic variation caused by exposure to environmental factors could also contribute to variable corrector responses *in vitro*. Measuring correction *in vitro* probably excludes some off-target drug effects, however, the underlying genetic and epigenetic factors that affect F508del-CFTR expression, folding and trafficking may persist.

We were interested to compare the variability of correction in cells from patients and in F508del-CFTR homozygous mice (van Doorninck et al., 1995; Wilke et al., 2011). The genomes and epigenomes of CF mice are expected to be more similar than humans because they are inbred and housed under identical conditions. We examined correction in the ileum because the CF phenotype is stronger in the intestine than in the lung in mice. In preliminary experiments we isolated small pieces of ileum from CF mice (*Cftr*^*tm*1*Eur*^) (van Doorninck et al., 1995) in quadruplicate and measured the forskolin-stimulated ΔI_sc_ after exposing tissues to DMSO or lumacaftor for 4 h *ex vivo* as described previously (Robert et al., 2010). Mixed, two-way ANOVA showed that the variability of F508del-CFTR correction was less in inbred mice than in patients. The interaction term (i.e., variability in drug response between individuals) accounted for ∼6.5% of the variance in mouse intestinal assays vs. 11.9% in HBE cultures from patients but was still significant (*P* < 0.0001). We believe this variation reflects the health of the mice or the condition of tissues after dissection, but cannot exclude that genetic and/or epigenetic variations persist in the mice despite inbreeding and the same environment.

The application of personalized medicine in CF has a bright future, however, the predictive value of *in vitro* assays remains to be established when there are alternative drugs and multiple patients carrying the same mutation. The present results indicate that patient-to-patient differences in F508del-CFTR correction can be assayed using airway cell cultures, however, a design effect due to group sampling needs to be compensated by increasing the number of replicates. Hopefully, CF correctors and corrector combinations will continue improving until they become so effective for all patients that precision medicine is no longer necessary (Hanrahan et al., 2017).

## Author Contributions

JH, EM, and DT: conceptualization. EM, JG, CM, JS, JL, and JH: investigation, and validation. JH: writing—original draft. JH, EM, JG, CM, and DT: writing—review and editing. JH and DT: funding acquisition. JG, CM, JL, and EM: resources.

## Conflict of Interest Statement

CF Translational Research Centre seminar series is supported by Vertex Pharmaceuticals Inc. JH is a mentor for the Vertex CF Research Innovation Award program. Vertex had no input into this study. The remaining authors declare that the research was conducted in the absence of any commercial or financial relationships that could be construed as a potential conflict of interest.

## Footnotes

1 www.graphpad.com

## References

[B1] Accurso F. J. Rowe S. M. Clancy J. P. Boyle M. P. Dunitz J. M. Durie P. R. (2010). Effect of VX-770 in persons with cystic fibrosis and the G551D-CFTR mutation. *N. Engl. J. Med.* 363 1991–2003. 10.1056/NEJMoa0909825 21083385 PMC3148255

[B2] Amaral M. D. (2015). Novel personalized therapies for cystic fibrosis: treating the basic defect in all patients. *J. Intern. Med.* 277 155–166. 10.1111/joim.12314 25266997

[B3] Blackman S. M. Commander C. W. Watson C. Arcara K. Strug L. J. Stonebraker J. R. (2013). Genome-wide association studies for type 2 diabetes and Cfrd reveal common risk loci. *Pediatr. Pulmonol.* 48 263–264.

[B4] Boyle M. P. Bell S. C. Konstan M. W. Mccolley S. A. Rowe S. M. Rietschel E. (2014). A CFTR corrector (lumacaftor) and a CFTR potentiator (ivacaftor) for treatment of patients with cystic fibrosis who have a phe508del CFTR mutation: a phase 2 randomised controlled trial. *Lancet Respir. Med.* 2 527–538. 10.1016/S2213-2600(14)70132-8 24973281

[B5] Brewington J. J. Filbrandt E. T. Larosa F. J. III Moncivaiz J. D. Ostmann A. J. Strecker L. M. (2018). Brushed nasal epithelial cells are a surrogate for bronchial epithelial CFTR studies. *JCI Insight* 3:98699. 10.1172/jci.insight.99385 29997283 PMC6124537

[B6] Burgener E. B. Moss R. B. (2018). Cystic fibrosis transmembrane conductance regulator modulators: precision medicine in cystic fibrosis. *Curr. Opin. Pediatr.* 30 372–377. 10.1097/MOP.0000000000000627 29538046 PMC6398332

[B7] Cholon D. M. Gentzsch M. (2018). Recent progress in translational cystic fibrosis research using precision medicine strategies. *J. Cyst. Fibros.* 17 S52–S60. 10.1016/j.jcf.2017.09.005 28986017 PMC5828944

[B8] Corvol H. Blackman S. M. Boelle P. Y. Gallins P. J. Pace R. G. Stonebraker J. R. (2015). Genome-wide association meta-analysis identifies five modifier loci of lung disease severity in cystic fibrosis. *Nat. Commun.* 6:8382. 10.1038/ncomms9382 26417704 PMC4589222

[B9] Cutting G. R. Kasch L. M. Rosenstein B. J. Zielenski J. Tsui L. C. Antonarakis S. E. (1990). A cluster of cystic fibrosis mutations in the first nucleotide- binding fold of the cystic fibrosis conductance regulator protein. *Nature* 346 366–368. 10.1038/346366a0 1695717

[B10] D’agostino R. B. Belanger A. D’agostin R. B. J. (1990). A suggestion for using powerful and informative tests of normality. *Am. Stat.* 44 316–321.

[B11] Dekkers J. F. Berkers G. Kruisselbrink E. Vonk A. De Jonge H. R. Janssens H. M. (2016). Characterizing responses to CFTR-modulating drugs using rectal organoids derived from subjects with cystic fibrosis. *Sci. Transl. Med.* 8:344ra384. 10.1126/scitranslmed.aad8278 27334259

[B12] Dekkers J. F. Wiegerinck C. L. De Jonge H. R. Bronsveld I. Janssens H. M. De Winter-De Groot K. M. (2013). A functional CFTR assay using primary cystic fibrosis intestinal organoids. *Nat. Med.* 19 939–945. 10.1038/nm.3201 23727931

[B13] Donner A. Klar N. (2000). *Design and Analysis of Cluster Randomization Trials in Health Research.* Oxford: Oxford University Press Inc.

[B14] Durmowicz A. G. Lim R. Rogers H. Rosebraugh C. J. Chowdhury B. A. (2018). The U.S. food and drug administration’s experience with ivacaftor in cystic fibrosis. establishing efficacy using *in vitro* data in lieu of a clinical trial. *Ann. Am. Thorac. Soc.* 15 1–2. 10.1513/AnnalsATS.201708-668PS 29020455

[B15] Eckford P. D. Ramjeesingh M. Molinski S. Pasyk S. Dekkers J. F. Li C. (2014). VX-809 and related corrector compounds exhibit secondary activity stabilizing active F508del-CFTR after its partial rescue to the cell surface. *Chem. Biol.* 21 666–678. 10.1016/j.chembiol.2014.02.021 24726831

[B16] Faul F. Erdfelder E. Lang A. G. Buchner A. (2007). G^∗^Power 3: a flexible statistical power analysis program for the social, behavioral, and biomedical sciences. *Behav. Res. Methods* 39 175–191. 10.3758/BF03193146 17695343

[B17] Flume P. A. Liou T. G. Borowitz D. S. Li H. Yen K. Ordonez C. L. (2012). Ivacaftor in subjects with cystic fibrosis who are homozygous for the F508del-CFTR mutation. *Chest* 142 718–724. 10.1378/chest.11-2672 22383668 PMC3435140

[B18] Fulcher M. L. Gabriel S. Burns K. A. Yankaskas J. R. Randell S. H. (2005). “Well-differentiated human airway epithelial cell cultures,” in *Human Cell Culture Protocols*, 2nd Edn, ed. Picot J. (New York, NY: Humana Press), 183–206. 10.1385/1-59259-861-7:183 15492373

[B19] Guimbellot J. S. Leach J. M. Chaudhry I. G. Quinney N. L. Boyles S. E. Chua M. (2017). Nasospheroids permit measurements of CFTR-dependent fluid transport. *JCI Insight* 2:95734. 10.1172/jci.insight.95734 29202459 PMC5752372

[B20] Hanrahan J. W. Matthes E. Carlile G. Thomas D. Y. (2017). Corrector combination therapies for F508del-CFTR. *Curr. Opin. Pharmacol.* 34 105–111. 10.1016/j.coph.2017.09.016 29080476

[B21] Killip S. Mahfoud Z. Pearce K. (2004). What is an intracluster correlation coefficient? Crucial concepts for primary care researchers. *Ann. Fam. Med.* 2 204–208. 10.1370/afm.141 15209195 PMC1466680

[B22] Martiniano S. L. Sagel S. D. Zemanick E. T. (2016). Cystic fibrosis: a model system for precision medicine. *Curr. Opin. Pediatr.* 28 312–317. 10.1097/MOP.0000000000000351 27031658 PMC4946574

[B23] Matthes E. Goepp J. Carlile G. W. Luo Y. Dejgaard K. Billet A. (2016). Low free drug concentration prevents inhibition of F508del CFTR functional expression by the potentiator VX-770 (ivacaftor). *Br. J. Pharmacol.* 173 459–470. 10.1111/bph.13365 26492939 PMC4728415

[B24] McCarthy C. Brewington J. J. Harkness B. Clancy J. P. Trapnell B. C. (2018). Personalised CFTR pharmacotherapeutic response testing and therapy of cystic fibrosis. *Eur. Respir. J.* 51:1702457. 10.1183/13993003.02457-2017 29563174

[B25] Motulsky H. J. (2014). *Intuitive Biostatistics. A Non-Mathematical Guide to Statistical Thinking.* New York, NY: Oxford University Press Inc.

[B26] Pranke I. M. Hatton A. Simonin J. Jais J. P. Le Pimpec-Barthes F. Carsin A. (2017). Correction of CFTR function in nasal epithelial cells from cystic fibrosis patients predicts improvement of respiratory function by CFTR modulators. *Sci. Rep.* 7:7375. 10.1038/s41598-017-07504-1 28785019 PMC5547155

[B27] Ratjen F. Bell S. C. Rowe S. M. Goss C. H. Quittner A. L. Bush A. (2015). Cystic fibrosis. *Nat. Rev. Dis. Primers* 1:15010. 10.1038/nrdp.2015.10 27189798 PMC7041544

[B28] Riordan J. R. (2008). CFTR function and prospects for therapy. *Annu. Rev. Biochem.* 77 701–726. 10.1146/annurev.biochem.75.103004.142532 18304008

[B29] Robert R. Carlile G. W. Liao J. Balghi H. Lesimple P. Liu N. (2010). Correction of the Delta phe508 cystic fibrosis transmembrane conductance regulator trafficking defect by the bioavailable compound glafenine. *Mol. Pharmacol.* 77 922–930. 10.1124/mol.109.062679 20200141

[B30] Robert R. Carlile G. W. Pavel C. Liu N. Anjos S. M. Liao J. (2007). Structural analogue of sildenafil identified as a novel corrector of the F508del-CFTR trafficking defect. *Mol. Pharmacol.* 73 478–489. 10.1124/mol.107.040725 17975008

[B31] Scholte B. J. Davidson D. J. Wilke M. De Jonge H. R. (2004). Animal models of cystic fibrosis. *J. Cyst. Fibros.* 3 183–190. 10.1016/j.jcf.2004.05.039 15463956

[B32] Sun L. Rommens J. M. Corvol H. Li W. Li X. Chiang T. A. (2012). Multiple apical plasma membrane constituents are associated with susceptibility to meconium ileus in individuals with cystic fibrosis. *Nat. Genet.* 44 562–569. 10.1038/ng.2221 22466613 PMC3371103

[B33] van Doorninck J. H. French P. J. Verbeek E. Peters R. H. Morreau H. Bijman J. (1995). A mouse model for the cystic fibrosis delta F508 mutation. *EMBO J.* 14 4403–4411. 10.1002/j.1460-2075.1995.tb00119.x 7556083 PMC394531

[B34] Van Goor F. Hadida S. Grootenhuis P. D. Burton B. Cao D. Neuberger T. (2009). Rescue of CF airway epithelial cell function *in vitro* by a CFTR potentiator, VX-770. *Proc. Natl. Acad. Sci. U.S.A.* 106 18825–18830. 10.1073/pnas.0904709106 19846789 PMC2773991

[B35] Van Goor F. Hadida S. Grootenhuis P. D. Burton B. Stack J. H. Straley K. S. (2011). Correction of the F508del-CFTR protein processing defect *in vitro* by the investigational drug VX-809. *Proc. Natl. Acad. Sci. U.S.A.* 108 18843–18848. 10.1073/pnas.1105787108 21976485 PMC3219147

[B36] Wilke M. Buijs-Offerman R. M. Aarbiou J. Colledge W. H. Sheppard D. N. Touqui L. (2011). Mouse models of cystic fibrosis: phenotypic analysis and research applications. *J. Cyst. Fibros* 10(Suppl. 2), S152–S171. 10.1016/S1569-1993(11)60020-9 21658634

[B37] Wright F. A. Strug L. J. Doshi V. K. Commander C. W. Blackman S. M. Sun L. (2011). Genome-wide association and linkage identify modifier loci of lung disease severity in cystic fibrosis at 11p13 and 20q13.2. *Nat. Genet.* 43 539–546. 10.1038/ng.838 21602797 PMC3296486

